# PP29 Social Preferences In Health Technology Assessments For Rare Diseases: A Systematic Literature Review Of New Analytic Approaches

**DOI:** 10.1017/S0266462324002022

**Published:** 2025-01-07

**Authors:** Paola Vasquez, Lisa Hall, Gregory Merlo

## Abstract

**Introduction:**

Rare diseases (RD) can be severe and can dramatically reduce life expectancy and quality of life. RD therapies, mostly orphan drugs (OD), fail to meet the standard criteria for public reimbursement due to uncertainty in cost-effectiveness estimates limiting healthcare access. To address this issue, experts have suggested the integration of social preferences into health technology assessments (HTAs) by implementing different methods.

**Methods:**

This systematic literature review, performed in 2021, aimed to explore worldwide experiences of social preferences integration into HTAs for RD and OD through the implementation of multiple-criteria decision analysis (MCDA), discrete choice experiments (DCE), and person trade-off (PTO), among other methods. A systematic search of the literature was conducted using PubMed, Cochrane, Embase, and Scopus databases. The PRISMA approach was used for the review phases. Finally, the Promoting Action on Research Implementation in Health Services (PARIHS) framework was used to discuss the implementation of these instruments in the RD context.

**Results:**

Thirty-three articles met the inclusion criteria. The studies measured social preferences for RD and OD as part of HTA using MCDA (n=17), DCE (n=8), and PTO (n=4), among other methods (n=4). These found that patients and clinicians do not prioritize funding based on rarity. The public is willing to allocate funds only if OD demonstrates effectiveness and improves the quality of life (QoL), considering as relevant factors disease severity, unmet needs, and QoL. Conversely, HTA agency experts preferred their current approach and placed more weight on cost-effectiveness and evidence quality even though they expressed concern about the fairness of the drug review process.

**Conclusions:**

MCDA, PTO, and DCE, among others, are helpful and transparent methods for assessing social preferences in HTAs for RD and OD. However, their methodological limitations, such as arbitrary criteria selection, subjective scoring methods, framing effects, weighting adaptation, and value measurement models, could be hurdles to implementation. Further research is needed to tailor these methods’ applicability and impact in different social contexts.

